# The association between sleep duration and physical performance in Chinese community-dwelling elderly

**DOI:** 10.1371/journal.pone.0174832

**Published:** 2017-03-30

**Authors:** Liyuan Fu, Liye Jia, Wen Zhang, Peipei Han, Li Kang, Yixuan Ma, Hairui Yu, Tianqi Zhai, Xiaoyu Chen, Qi Guo

**Affiliations:** 1 Department of Rehabilitation Medicine, Cardiovascular Clinical College of Tianjin Medical University, TEDA International Cardiovascular Hospital, Tianjin, China; 2 Department of Rehabilitation and Sports Medicine, Tianjin Medical University, Tianjin, China; Indiana University Richard M Fairbanks School of Public Health, UNITED STATES

## Abstract

**Background:**

Physical performance is an important healthy factor in elder people. Good living habits, which include sleep, can maintain physical strength and physical performance. The aim of the present study was to conduct a cross-sectional study to determine the association between total sleep duration and physical performance.

**Methods:**

Our study population comprised residents of the township central hospital in the suburban of Tianjin, China. We measured muscle strength, walk speed and balance function by grip, 4-m walk test and timed up and go test (TUGT). We divided sleep duration into four groups <7h, 7-8h, >8-9h, >9h.

**Results:**

A total 898 participants had completed data (392 men and 506 women, mean age 67.71 years). In man, adjusted sleep duration was associated with lower grip in > 9 h group, the mean value (95% CI) was 0.429 (0.409, 0.448), and longer TUGT time was also associated with long sleep duration, 10.46s (9.97 s, 10.95 s). In women, adjusted slower 4-m walk speed present an inverse U-shaped relation with sleep duration, by 0.93 m/s (0.86 m/s, 0.98 m/s), 0.97 m/s (0.96 m/s, 1.00 m/s), 0.97 m/s (0.95 m/s, 0.99 m/s) and 0.92 m/s (0.89 m/s, 0.96 m/s); longer TUGT time were associated with long sleep duration (> 9 h), by 11.23 s (10.70 s, 11.77 s).

**Conclusion:**

In Chinese community-dwelling elderly, lower muscle strength and lower balance function were associated with long sleep duration in men. Slower walk speed and lower balance function were associated with long sleep duration in women.

## Introduction

Physical performance is an important healthy factor in elder people. As the growth of the age, physical performance such as balance, walk speed, muscle strength diminished [1], leading to high risks of falls and subsequent fractures, even lose independence, increase morbidity and mortality rates [2–4]. Thus, it is essential to improve or prevent the factors of physical performance decline in elderly at once. Good living habits, which may be one of ways to solve this problem, had been paid more and more attention by the human beings. While in recent years, as an important component of living habits, sleep is becoming a significant problem for elder people.

Epidemiological studies report that more than half of people who over the age of 65 years suffer from variable sleep problems [5]. Though sleep may maintain physical strength and physical performance, people over 60 years showed a U-shape in all-cause mortality, cardiovascular disease (CVD) mortality, respiratory disease mortality with sleep duration [6]. Furthermore, growing evidence indicates that long sleep duration may increase the risk of disease [7–9] and all-cause mortality, and decline the self-related health [10, 11], especially in elderly. So the sleep effect on elderly health remains to need more researches.

So we have reason to believe that it does exist a relation between sleep duration and physical function, which influence the morbidity and mortality of disease. Recently, a little studies have examined the association between sleep duration and physical performance. Some studies show that short[12, 13] or long sleep duration[14, 15] may have negative effect on physical performance, but there haven’t reached an agreement on the subcomponent of physical performance with sleep duration, especially in elderly people [12–17]. Moreover, the potential mechanism(s) that could explain the relationship with long sleep duration is unclear. Thus, more attention should be given to the long sleep duration.

Therefore, the aim of the present study was to conduct a cross-sectional study to determine the association between total sleep duration and physical performance among Chinese community-dwelling elderly individuals, which will provide reference for predicting decreased physical performance and find out high falls risk of people to decrease the mortality. According to these, we can take intervention or rehabilitation at once to reduce physical performance decline, provide a guide to healthy living habits. We selected three indicators, grip, TUGT and 4-m walk speed to measure the muscle strength and gait function (balance and walk speed).

## Method

### Participants

Our study population comprised residents of the township central hospital of Chadian, in the suburban Hangu of Tianjin, China. From March to April 2013 and July to August 2015, participants who joined the national free physical examination programs were aged ≥60 years old. All subjects were invited to participate in a comprehensive geriatric assessment, with the exception of those with a disability that affected the basic activities of daily living, and thus could not carry out performance-based assessments.

997 older individuals (age ≥60) joined the examination program from March 2013 to August 2015. The exclusion criteria were as follows: (1) age < 60 years, (2) serious arthropathic deformation of knee joint causing severe mobility impairment or localized loss of strength, (3) Parkinson’s disease(Hoehn-Yahr III-V), serious illness interfering with the conduct of the study or interpretation of the results, (4) current use of androgens or antiandrogens, (5) visual disorder with no appropriate clinical correction with corrective lenses at the time of the tests, (6) a specific test (such as TUGT, 4-meter walking test and so on) if they could not perform it independently or safely, based on the judgment of the geriatric nurse or subjects themselves, (7) medical record diagnosis of dementia and other memory system disease, (8) not fixed time for sleep or day and night sleep upside down, and (9) refusal to participate the follow-up in our cohort study. A total of 898 subjects were included in follow-up group. We excluded 2 participants who were not competent to give informed consent, 1 who did not perform handgrip strength because of arthritis in her fingers, 3 participants who could not perform 4-m walking test because of visual disorder, 1 participant who was diagnosed dementia and 2 participants who did not undergo bioelectrical impedance analysis (BIA) because of a pacemaker. Participants provided their written informed consent to participate in this study. This study was approved by ethics committee at Tianjin Medical University, China.

### Performance-based assessment

Performance-based assessment consisted of several physical tests and muscle mass. We have described the methods of 4-m walk tests, TUGT, grip strength in detail in our previous study [18]. Grip (kg) was used as a measure of muscle strength and was quantified using a handheld dynamometer (GRIP-D; Takei Ltd, Niigata, Japan). Participants were asked to exert their maximum effort twice using their dominant hand and the average grip strength was recorded. Gait function was assessed with the 4-m walk tests. We use 4-m walk speed for measuring the speed that people walk, and TUGT was used to test balance function, which is also frequently used to assess fall risk in older adults [19]. To measure walking speed, two photocells connected to a recording chronometer were placed at the beginning and the end of a 4-meter course at the site clinic. Participants were instructed to stand with both feet touching the starting line and to begin walking at their usual pace after a verbal command was given. The time between activation of the first and the second photocell was measured and the average speed of two walks was recorded. The TUGT involved rising from a chair, walking for 3 meter, turning around, walking back to the chair, and sitting down. The time taken at the participant’s usual pace was measured in seconds once. To avoid the measurement error, the assessment was conducting by postgraduate students in the health field who received special training for testing administered all tests as part of a standard geriatric assessment. And each item shall be the responsibility of the personnel. Every project is respectively by the same trained staff to complete the data collection of all the subjects. Muscle mass was measured using a direct segmental multi-frequency BIA (In-Body720; Biospace Co., Ltd, Seoul, Korea).

### Assessment of sleep behavior

Self-reported sleep duration was measured by trained interviewers as a component of the interview. Participants were asked the time they usually went to bed and got up during the past month, which is same with some previous studies [15, 20, 21]. We calculated the time they slept per night. We partially reference the standard of general method of cutting sleep duration, which dividing persons into short sleepers (< 7 h), long sleepers (≥ 9 h), and mid-range sleepers were divided into two groups (7–8 h, > 8–9 h; one hour per group) [22, 23]. Wake after sleep onset (WASO), a measure of fragmented sleep, calculated as the average number of hours awake between sleep onset (where sleep onset was scored as the completion of 20 continuous minutes of sleep after getting into bed) and final awakening.

Sleep quality was inquired by asking subjects “Do you think you slept enough during the past month?” (People have 4 choices: 1 = very well 2 = good 3 = not enough 4 = very poor). We also asked subjects whether they took sleeping pills[24](include Eszopiclone, Zaleplon, Zolpidem, Melatonin, Ramelteon, Benzodiazepine Hypnotics, Antidepressants, Suvorexant et,al) before they went to bed.

### Covariates

Data regarding sociodemographic, behavioral characteristics, and physical illness of dates were obtained as previously (via face-to-face questions). Sociodemographic variables, including age, gender, marital status, educational level, and occupation, were assessed. Marital status was classified as married (living together, divorced, separated or widowed) or not married/single. Educational level was defined as age at completion of schooling and divided into 4 categories: illiterate, 1–6 y, 7–12 y and ≥13 y. Behavioral characteristics included smoking and drinking habits. Information on smoking (never, former smoker, or current smoker) and drinking (never, former drinker, occasional drinker, or everyday drinker) was also obtained from the questionnaire. Physical activity was assessed using the short form of the International Physical Activity Questionnaire (IPAQ). We have described the methods of IPAQ in detail in a previous study [18]. Depressive symptoms were assessed using the Geriatric Depression Scale (GDS).

### Statistical analysis

Differences between variables were examined by ANOVA with Bonferroni correction on continuous variables. And we use the chi square test on categorical variables with sleep duration group. Data are presented as means (with 95% confidence intervals) or as percentages. Linear regression analysis (ANCOVA) was used to assess whether participants’ sleep duration associated with physical performance. Covariates were added sequentially to the linear model to evaluate association at different levels of adjustment. Crude was unadjusted, Model 1 was adjusted for age. Model 2 was additionally adjusted for BMI, widow, live alone, diabetes, falls, skeletal muscle mass and IPAQ on the base of Model 1. Model 3 additionally adjusted for sleep quality, sleep drug taking on the base of Model 2. Differences were defined as significant when P<0.05. All statistical analyses were performed with the SPSS V19.0 software package (SPSS Inc, China).

## Results

### Subject characteristics

A total 898 participants (392 men and 506 women) aged 60 to 93 had complete data in 2013–15 (the baseline of the present study) and were included in the analysis. Tables 1 and 2 show the baseline characteristics of the men and women, mean age of these two groups were 68.07 (0.529) and 67.36 (0.498). Of these participants, the numbers of subjects who slept for <7h, 7-8h, >8-9h, >9h in men were 43, 152, 112, 85 subjects. And in women were 40, 217, 155, 94 subjects, separately. In women, widowed people in group <7h and >9h were 21.6% and 28.0%, higher than 7-8h group. In <7h, >8-9h, >9h group, women who experience falls were 17.5%, 14.3%, 24.5%, higher than mid-range sleep duration.

**Table 1:** Subject characteristics according to gander and categories of sleep duration.---Male.

	Sleep Duration				P
	<7	7–8	>8–9	>9	
N	43	152	112	85	
Age, y	68.00 (0.99)	67.18 (0.52)	67.62 (0.59)	69.46 (0.81)	0.083
Height, cm	171.72 (0.86)	170.01 (0.51)	169.33 (0.66)	170.59 (0.68)	0.184
Weight, kg	75.65 (1.37)	72.35 (0.85)	72.03 (0.95)	73.27 (1.09)	0.210
BMI, kg/m^2^	25.6 (3.83)	25.0 (2.65)	25.1 (2.93)	25.2 (3.24)	0.701
Live alone, %	25.6	17.8	9.8	11.8	0.052
Widow, %	14.0	8.6	6.3	12.9	0.321
Education, %					0.169
Illiteracy	9.3	13.2	19.6	21.2	
Non-illiteracy	90.7	86.8	80.4	78.8	
Farmer, %	74.4	80.9	79.5	77.6	0.816
Nonsmoker, %	41.9	49.3	33.9	34.1	0.201
Nondrinker, %	37.2	33.6	28.6	37.6	0.147
Sleep quality,%					0.051
Very well	48.5	44.7	46.4	58.8	
Good	25.6	38.8	37.5	20.0	
Not enough	7.0	7.2	10.7	8.2	
Very poor	18.6	9.2	4.5	12.9	
Sleep drug taking	9.3	7.9	8.0	9.4	0.973
IPAQ, Met/week	5279 (959)	4496 (518)	5121 (738)	3864 (684)	0.551
Grip, kg/kg	0.466 (0.012)	0.473 (0.008)	0.474 (0.008)	0.419 (0.011)	<0.001
TUGT, s	9.22 (0.27)	9.68 (0.27)	9.66 (0.18)	10.88 (0.42)	0.005
Walk speed, m/s	1.04 (0.025)	1.05 (0.015)	1.05 (0.019)	1.00 (0.027)	0.389
Skeletal muscle mass, kg	23.93 (0.49)	22.42 (0.29)	22.04 (0.31)	22.32 (0.37)	0.021
Disease, %					
Diabetes	11.6	9.2	10.7	8.2	0.905
Hypertension	39.5	44.1	43.8	35.3	0.559
Hyperlipidemia	27.9	38.8	42.0	44.7	0.299
Falls, %	16.3	12.5	7.1	18.8	0.091
GDS, %					0.593
Normal	90.7	95.4	92.9	91.8	
Depression	9.3	4.6	7.1	8.2	

BMI, body mass index; IPAQ, international physical activity questionnaire; TUGT, timed up and go test.

Mean; Standard error in parentheses (all such values).

Obtained by using ANOVA for continuous variables and chi-square for variables of proportion.

**Table 2:** Subject characteristics according to gander and categories of sleep duration----Female.

	Sleep Duration				P
	<7	7–8	>8–9	>9	
N	40	217	155	94	
Age, y	68.48 (0.88)	65.42 (0.34)	67.15 (0.48)	68.40 (0.67)	<0.001
Height, cm	157.36 (0.99)	157.81 (0.44)	157.45 (0.50)	156.05 (0.69)	0.173
Weight, kg	64.82 (1.91)	63.56 (0.77)	62.28 (0.80)	62.29 (1.17)	0.439
BMI, kg/m^2^	26.1 (6.41)	25.5 (2.85)	25.1 (2.82)	25.5 (4.03)	0.482
Live alone, %	27.5	16.1	9.0	14.9	0.022
Widow, %	21.6	13.5	13.2	28.0	0.008
Education, %					0.082
Illiteracy	37.5	31.8	28.4	43.6	
Non-illiteracy	62.5	68.2	71.6	56.4	
Farmer, %	85.0	88.0	92.9	94.7	0.120
Nonsmoker, %	32.5	28.6	33.5	23.4	0.195
Nondrinker,%	85.0	90.8	90.3	91.5	0.524
Sleep quality,%					0.011
Very well	20.0	43.3	41.3	38.3	
Good	30.0	32.3	29.7	26.6	
Not enough	20.0	12.9	12.9	8.5	
Very poor	30.0	11.5	16.1	26.6	
Sleep drug taking	40.0	14.7	14.2	17.0	0.001
IPAQ, Met/wk	3094 (610)	3083 (317)	3027 (490)	2584 (292)	0.715
Grip, kg/kg	0.311 (0.011)	0.328 (0.006)	0.318 (0.007)	0.303 (0.009)	0.120
TUGT, s	10.41 (0.53)	9.78 (0.18)	10.37 (0.22)	11.65 (0.35)	<0.001
Walk speed, m/s	0.91 (0.032)	0.99 (0.012)	0.95 (0.014)	0.89 (0.020)	<0.001
Skeletal muscle mass, kg	16.28 (0.51)	16.34 (0.36)	15.94 (0.23)	15.18 (0.35)	0.132
Disease, %					
Diabetes	2.5	11.1	23.2	13.8	0.001
Hypertension	42.5	50.2	46.5	54.3	0.524
Hyperlipidemia	45.0	50.2	52.9	62.8	0.151
Falls, %	17.5	14.3	24.5	33.0	0.002
GDS, %					0.817
Normal	82.5	89.9	89.0	88.3	
Depression	17.5	10.2	10.9	11.7	

BMI, body mass index; IPAQ, international physical activity questionnaire; TUGT, timed up and go test.

Mean; Standard error in parentheses (all such values).

Obtained by using ANOVA for continuous variables and chi-square for variables of proportion.

### Physical performance

Table 3 shows the mean (95%CI) of 4-m walk tests, TUGT, grip for 4 groups of sleep duration in men. With the reference of the 7-8h group, no significant associations between 4-m walk speed and sleep duration was found. After adjustment, sleep duration was associated with lower grip in >9h group, 0.429 (0.409,0.448). Adjusted TUGT was associated with long sleep duration, 10.46 s (9.97 s, 10.95 s).

**Table 3:** Unadjusted and adjusted modal for sleep duration with physical performance in male.

Physical Performance	Sleep Duration				P^1^
	<7	7–8	>8–9	>9	
**N**	43	152	112	85	
**Walk speed, m/s**					
Crude	1.04 (0.98,1.11)	1.05 (1.02,1.08)	1.05 (1.01,1.09)	1.01 (0.96,1.05)	0.389
Model1^2^	1.05 (0.99,1.10)	1.04 (1.01,1.07)	1.05 (1.01,1.08)	1.03 (0.98,1.07)	0.873
Model2^3^	1.06 (0.99,1.12)	1.05 (1.03,1.08)	1.06 (1.02,1.09)	1.04 (0.99,1.07)	0.953
Model3^4^	1.06 (0.99,1.12)	1.05 (1.03,1.08)	1.05 (1.02,1.09)	1.04 (0.99,1.07)	0.972
**TUGT, s**					
Crude	9.22 (8.32,10.12)	9.68 (9.20,10.16)	9.66 (9.11,10.22)	10.88 (10.25,11.52) *^5^	0.005
Model1^2^	9.19 (8.41,9.98)	9.84 (9.42,10.26)	9.73 (9.24,10.21)	10.53 (9.97,11.09) *	0.035
Model2^3^	9.35 (8.65,10.02)	9.73 (9.25,10.13)	9.74 (9.36,10.18)	10.47 (9.97,10.99) *	0.027
Model3^4^	9.32 (8.62,10.03)	9.69 (9.25,10.10)	9.75 (9.33,10.17)	10.46 (9.97,10.95) *	0.021
**Grip, kg/kg**					
Crude	0.466 (0.437,0.496)	0.472 (0.457,0.488)	0.474 (0.456,0.493)	0.419 (0.398,0.440) *	<0.001
Model1^2^	0.467 (0.439,0.495)	0.469 (0.454,0.485)	0.473 (0.456,0.491)	0.427 (0.407,0.447) *	0.003
Model2^3^	0.469 (0.447,0.492)	0.471 (0.452,0.483)	0.472 (0.455,0.487)	0.429 (0.409,0.448) *	0.007
Model3^4^	0.466 (0.443,0.491)	0.470 (0.452,0.482)	0.471 (0.454,0.488)	0.429 (0.409,0.448) *	0.005

^1^ Obtained by using ANCOVA;

^2^ Adjusted for age;

^3^ Additionally adjusted for BMI; widow; live alone; diabetes; falls; skeletal muscle mass; IPAQ on the base of Model 1.

^4^ Additionally adjusted for sleep quality; sleep drug taking on the base of Model 2.

^5^ Mean; 95% CI in parentheses (all such values).

* compare with 7-8h sleep duration group (P<0.05).

Table 4 shows the mean (95%CI) of 4-m walk tests, TUGT, grip for 4 groups of sleep duration in women. With the reference of the 7-8h group, 4-m walk speed was significantly difference between < 7 h, > 8–9 h, > 9 h group, and it presents an inverse U-shaped with sleep duration, by 0.91 m/s (0.85 m/s, 0.96 m/s), 0.99 m/s (0.97 m/s, 1.02 m/s), 0.96 m/s (0.93 m/s, 0.98 m/s), 0.89 m/s (0.86 m/s, 0.93 m/s). After adjusted, the trend still exists, but only slower walk speed was associated with >9h sleep duration, 0.92 m/s (0.89 m/s, 0.96 m/s). After adjustment for significant variables, sleep duration was associated with long TUGT in > 9 h group, 11.23 s (10.70 s, 11.77 s). No significant associations between grip and sleep duration in women.

**Table 4:** Unadjusted and adjusted modal for sleep duration with physical performance in female.

Physical Performance	Sleep Duration				P^1^
	<7	7–8	>8–9	>9	
**N**	40	217	155	94	
**Walk speed, m/s**					
Crude	0.91 (0.85,0.96) *	0.99 (0.97,1.02)	0.96 (0.93,0.98) *	0.89 (0.86,0.93) *^5^	0.043
Model1^2^	0.93 (0.88,0.98) *	0.98 (0.95,0.99)	0.96 (0.93,0.99)	0.92 (0.88,0.95) *	0.017
Model2^3^	0.92 (0.84,0.99) *	0.96 (0.96,1.00)	0.98 (0.96,0.99)	0.92 (0.89,0.95) *	0.016
Model3^4^	0.93 (0.86,0.98) *	0.97 (0.96,1.00)	0.97 (0.95,0.99)	0.92 (0.89,0.96) *	0.024
**TUGT, s**					
Crude	10.41 (9.52,11.31)	9.79 (9.40,10.17)	10.37 (9.92,10.83)	11.65 (11.07,12.24) *	0.013
Model1^2^	10.05 (9.23,10.88)	10.06 (9.71,10.42)	10.29 (9.87,10.71)	11.31 (10.77,11.85) *	0.002
Model2^3^	10.34 (9.56,11.13)	9.98 (9.67,10.39)	10.20 (9.79,10.66)	11.29 (10.72,11.78) *	0.003
Model3^4^	10.32 (9.52,11.15)	9.96 (9.60,10.31)	10.18 (9.74,10.52)	11.26 (10.70,11.77) *	0.004
**Grip, kg/kg**					
Crude	0.311 (0.284,0.339)	0.328 (0.316,0.340)	0.318 (0.340,0.332)	0.303 (0.285,0.320) *	0.120
Model1^2^	0.319 (0.292,0.345)	0.322 (0.311,0.334)	0.320 (0.306,0.333)	0.310 (0.292,0.327)	0.688
Model2^3^	0.311 (0.284,0.339)	0.325 (0.313,0.336)	0.324 (0.308,0.338)	0.312 (0.294,0.330)	0.543
Model3^4^	0.314 (0.286,0.341)	0.325 (0.314,0.337)	0.323 (0.310,0.337)	0.310 (0.293,0.330)	0.523

^1^ Obtained by using ANCOVA;

^2^ Adjusted for age;

^3^ Additionally adjusted for BMI; widow; live alone; diabetes; falls; skeletal muscle mass; IPAQ on the base of Model 1.

^4^ Additionally adjusted for sleep quality; sleep drug taking on the base of Model 2.

^5^ Mean; 95% CI in parentheses (all such values).

* compare with 7-8h sleep duration group (P<0.05).

## Discussion

Our findings suggest a difference relationship between sleep duration and physical performance in men and women. We adjusted the age, widow, live alone, diabetes, cardiovascular risk, falls, skeletal muscle mass, sleep quality and sleep drug long sleep duration was best associated with slower walk speed, longer TUGT in women and longer TUGT, lower grip in men. We found that there might be contrary relation between long sleep duration and balance function (measured by TUGT) in both men and women. This is an important finding because poor physical performance always accompany high risk of morbidity and mortality of disease [3, 4]. To our knowledge, this is the first studies to explore the different relationship in sleep duration and physical performance by gender variance in Chinese community-dwelling elderly.

### Sleep duration and TUGT

Our study found that both men and women who slept over 9 hours showed poorer balance function than those slept for 7–8 hours. It also seems that elder people who had long sleep duration were more probably have lower balance function in previous studies [16, 25] It may explained by that long sleep duration give rise to an inverted relationship with muscle mass[26] and lower skeletal muscle mass content has been reported to be associated with poor functional capacities, which could lead to an increase risk for falls and disabilities [27, 28]. In the present study, we noted that skeletal muscle mass in long sleepers (>9h group) were significantly lower than mid-range sleepers (7-8h group) in men (22.32 kg VS 22.42 kg, p = 0.019, ANOVA) and women (15.18 kg VS 16.34 kg, p = 0.025, ANOVA), which could be potential mechanisms in explaining the poorer performance in balance among long sleepers. The level of skeletal muscle mass is different from skeletal muscle strength, especially in postmenopausal women. Muscle strength appears to decline more with age than muscle mass [29, 30]. So long sleep duration may be associated with balance but not grip in women. While this result is different with some other studies, Reyes [12] found short sleep duration was associated with functional performance impairment but not the long sleep duration. This may because the mean age of this study was older than the mean age in present study, lead to lower balance function in short sleepers. Moreover, this study included >8.5h in long sleep group, confused the sleep positive effect on grip in this results. While in the InCHIANTI study[15], balance function wasn’t associated with long sleep duration but associated with time in bed. This is an important finding because they separated time in bed from the sleep duration, to clearly interpret the relation between balance function and true (objective) sleep time or time in bed. Our study only measured subjective sleep duration, it may contain ‘time in bed’, which made the relationship significant. So further study need explore the to explore the effect of true sleep duration on physical performance. As well, individuals who slept more than 9 hours per day had 15% less physical activity per week than those sleeping 7 or 8 hours per day[31]. In present study, we also found that trends in physical activity with long sleepers compared with mid-range sleepers both older men and women (3864 Mets/wk vs 4496 Mets/wk for men; 2584 Mets/wk vs 3083 Mets/wk for women). People may gain poor physical performance from the less physical activity because of the long sleep duration.

### Sleep duration and grip

Long sleep duration may has lower grip for less physical activity, high fibrinogen, rapid eye movement sleep (REM). This cross-sectional study showed that long sleep duration was associated with lower grip in men, but no relationship in women. This result is consistent with findings found in sleep studies for men [14] and women [16]. Long sleep duration might make negative effect on grip by cardiovascular risk and mortality [32, 33] and high fibrinogen that may lead to coronary heart disease[34],but the mechanism hasn’t found. In our study, we found that male long sleepers’ cardiovascular risk (21.2%) is higher than mid-range sleepers (20.4%). This relation different with Reyes’ study [12], it might because there were 77% subjects were women, almost take major in the subjects, mixed the results in grip. Moreover, this “long sleep duration” may not true long, which include >8.5h in long sleep group, confused the sleep positive effect on grip in this results. So as to take account for no relation between strength and long sleep duration.

In women, a longer sleep time may create less of an opportunity to engage in physical activity. In experimental studies, extended bed rest, irrespective of sleeping, is shown to accompany a marked decrease in muscle strength [35] as well as insulin resistance and cardiac atrophy [36], linking long sleep duration to low physical performance. While, it is different with some other studies in women. In a postmenopausal women study [25], long sleep duration had inverse relation with muscle strength, it might because the middle-aged adults take account for the significance in this study (49-85y).

The decreasing amount of rapid eye movement sleep (REM) was associated with lower grip and slower walk speed in men [14], but the real mechanism hasn’t found. Due to the lack of estrogen, the skeletal muscle strength in postmenopausal women declined[37], and lower than men in total. The estrogen factor may confusion the results for grip in relation to the long sleep duration in women.

We have found that good relation of long sleep duration with lower grip strength, further study we need to explore the mechanism of this relation.

### Sleep duration and walk speed

Our study showed that long sleep duration was significantly related to slower usual walk speed in women but not in men, which has similar results in the some other studies [15]. We can find that sleep duration showed a U-shaped relation with walk speed in women in crude, model 1 and model 2. But after adjusted sleep quality and sleep drug taking, this relationship didn’t exist anymore. It may indicate that poorer sleep quality is the key factor on the relation between short sleep duration and slower walk speed in older women. So we couldn’t conclude that walk speed show a U-shaped association with sleep duration. Goldman [16] also studied the relation between sleep duration with walk speed, slower walk speed was associated with short sleep duration but not long sleep duration. One reason is the study didn’t exam the relation between long sleep duration; the other reason is that they didn’t exclude the effect of sleep quality on this relation. Long sleep duration may bring higher risk of osteoporosis in post-menopausal women than nocturnal sleepers [38]. Osteoporosis is related to poor physical function, especially slower walk speed. Moreover, the pain of osteoporosis leads to fear of falling [39], these conditions may take account for slower walk speed even lower TUGT time. Our data showed those 21.1% male long sleepers and 35.1% female long sleepers reported poor sleep quality. We take sleep quality into account in the relationship of sleep duration and physical performance. The relationship between long sleep duration and physical performance didn’t change before and after adjusting sleep quality. We can conclude that however sleep quality is, long sleep duration have negative effect on physical performance. Perhaps they couldn’t benefit from their long sleep duration due to their tiredness and fatigue[40]. This may indicate why self-reported long sleep duration is associated with failure restorative functions from sleep [41].

Whereas we haven’t found the relation between sleep duration and walk speed in men, which is different from Dam’s study[14]. Age-adjusted slower walk speed was associated with long sleep duration, after multivariate adjusted the relation didn’t exist; but in the association between sleep measures and binary physical function outcomes, short sleep duration was associated with slower walk speed, not long sleep duration. Perhaps this long sleep duration (≥8h) include the mid-range sleep, which mix the positive effect in to this relation.

We have found that good relation of long sleep duration with slower walk speed in older women, which might related to poor physical performance to intervene correcting unhealthy sleep habits.

### Strength and limitations

Our study has provided a reference to suggest that long sleep duration is associated with poor physical performance, which may provide a reference for clinical medicine for predicting the presenting of poor physical performance in elderly. This study also has some limitations. The analysis subset was community-dwelling elderly in Tianjin, China, and may not be representative of the general elderly. Though we used self-reported method to measure the sleep duration, it is also an convenient way for large-sample studies, especially for large-scale cohort study. It does not as accurately as actigraph-measured sleep duration. While, previous study has shown that self-reported sleep duration moderately correlated with actigraph-measured sleep[42]. Finally, this was a cross-sectional study. Though our result indicate that there is relation between short/long sleep duration and poor physical performance, we could not conclude whether short /long sleep duration led to an increase in the occurrence of physical dysfunction. While, this result might help health care providers identify elderly people in which interventions to improve sleep may reduce risk for disability. Longitudinal studies are needed to determine if long sleep durations are causally associated with functional decline.

## Conclusion

Our study found that lower grip was associated with long sleep duration in men, and walk speed and lower balance were associated with long sleep duration in women. Given the prevalence of long sleep duration in older adults, our findings suggest that interventions of sleep may reduce physical functional decline among the rising population of older adults. Future research with cohort study should test whether long sleep durations are causally associated with functional decline and the effect that intervention of sleep on physical performance among community-dwelling older adults.
